# Tuberculous septic arthritis of the hip with large abscess formation mimicking soft tissue tumors: A case report

**DOI:** 10.1016/j.heliyon.2021.e06815

**Published:** 2021-04-17

**Authors:** Marcel Prasetyo, Indah Maria Adistana, Stefanus Imanuel Setiawan

**Affiliations:** Department of Radiology, Faculty of Medicine Universitas Indonesia – Dr. Cipto Mangunkusumo National Central General Hospital, Jakarta, Indonesia

**Keywords:** Tuberculosis, Septic arthritis, Infectious arthritis, Abscess

## Abstract

**Background:**

Tuberculous septic arthritis is an infection that occurs inside the joint or synovial fluid and joint tissues caused by *Mycobacterium tuberculosis*. It may show wide variability of clinical symptoms and imaging appearance, ranging from asymptomatic with a normal radiographic examination to severe joint pain along with joint destruction, osteomyelitis, and abscess formation. This article presents radiographic and MR imaging appearance from a case of tuberculous septic arthritis with large abscess formation mimicking soft tissue tumor.

**Case presentation:**

We reported a 32-year-old female with a slowly enlarging lump on her right proximal thigh within the last 4 months along with slowly progressing joint pain. Both radiographic and MR images showed destruction of the femoral head and acetabular roof, with a formation of large rim-enhanced abscess that extending superficially and distally until mid-thigh. The patient underwent open drainage surgery and excisional biopsy. Histopathological examination showed chronic granulomatous inflammation caused by tuberculous infection.

**Conclusion:**

MR imaging combined with radiographic and clinical information played a very important role in the diagnosis of tuberculous septic arthritis with abscess, and to differentiate it from soft tissue neoplasms.

## 1 Introduction

Musculoskeletal involvement in tuberculous disease is an uncommon manifestation of extrapulmonary tuberculosis [1]. In Indonesia, the Minister of Health found that from a total of 563,987 confirmed tuberculous cases, about 59,525 (11%) cases were extrapulmonary tuberculosis in 2019 [2]. Bone and joint involvement account for 9% of extrapulmonary tuberculosis with spine, knee and hip joints as the most commonly affected peripheral joint. About 15–20% of the tuberculous septic arthritis cases involve the hip joints [3]. Tuberculous septic arthritis progresses slowly and chronically which makes the patient showing subtle symptoms and imaging abnormalities lead to delayed diagnosis [[4], [5]]. It may also form a large tumor-like abscess that is easily misdiagnosed as a soft tissue tumor lesion. This article will focus on the imaging appearance of tuberculous septic arthritis and how to differentiate it from pyogenic septic arthritis and soft tissue tumor.

## 2 Case presentation

**Figure 1:**
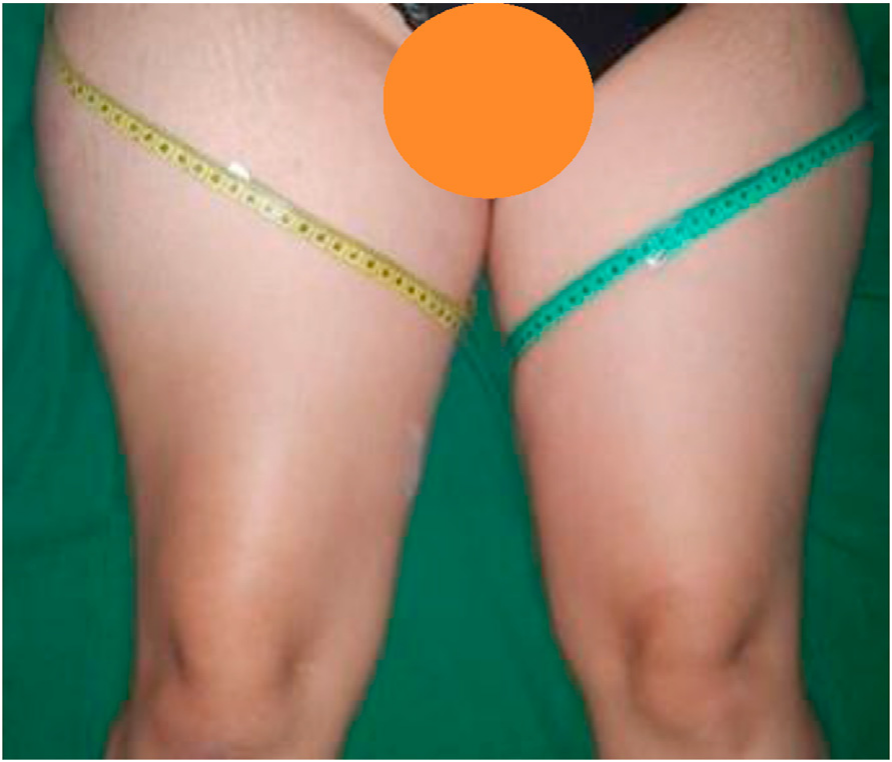
The clinical presentation of the patient. Soft tissue enlargement of the right proximal femoral region compared to the normal left thigh with no discoloration and inflammation sign was observed. The patient experienced mild tenderness that slowly progressed within 4 months.

We reported a 32-year-old female patient who came to the hospital after being referred from another hospital to have further treatment for a soft tissue tumor of her right thigh. She complained of a slowly enlarging lump on her right proximal thigh in the last 4 months, with intermittent sub febrile temperature and mild but slowly progressing pain. She was unable to walk unsupported. Physical examination showed a large fusiform soft tissue mass in the right thigh with a diameter of 84 cm, limited range of motion, moderately firm in palpation, and tenderness of the right hip (Figure 1). In the previous hospital, the patient underwent a hip radiograph which showed a large soft tissue mass on the right hip and was assessed as a soft tissue tumor. Written informed consent was obtained from the patient for publication of this case report and the patient's identity was being kept anonymous.

### 2.1 Investigations

**Figure 2:**
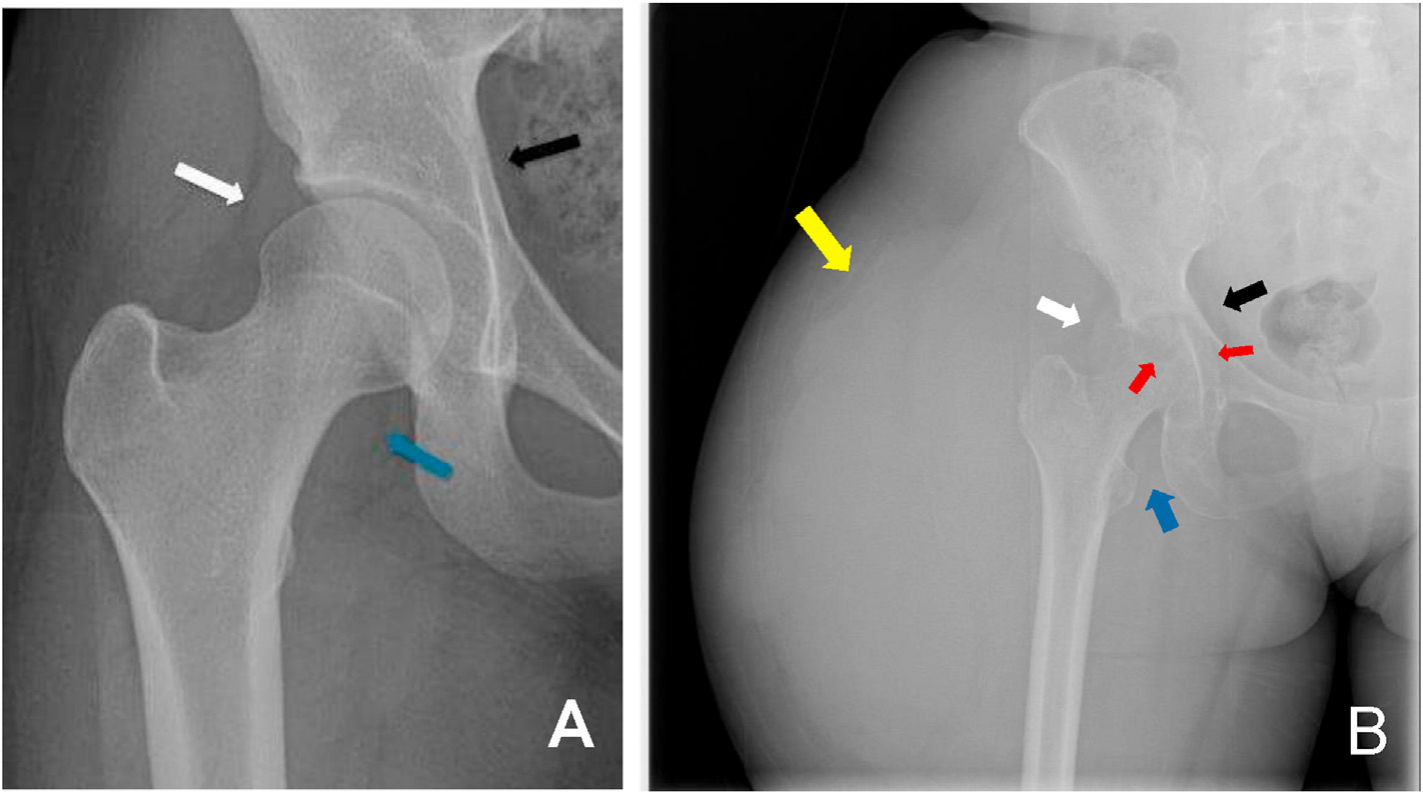
(A) Normal hip radiograph shows normal gluteal fat line (white arrow), iliopsoas fat line (blue arrow), and obturator fat line (black arrow). (B) The patient's right hip radiograph shows a slightly blurred gluteal fat line (white arrow) and obturator fat line (black arrow), with indistinct iliopsoas fat line (blue arrow). A large lobulated soft tissue mass at the lateral side of the femur (yellow arrow) with the destruction of femoral head and acetabular wall (red arrows), consistent with severe central erosion was observed.

**Figure 3:**
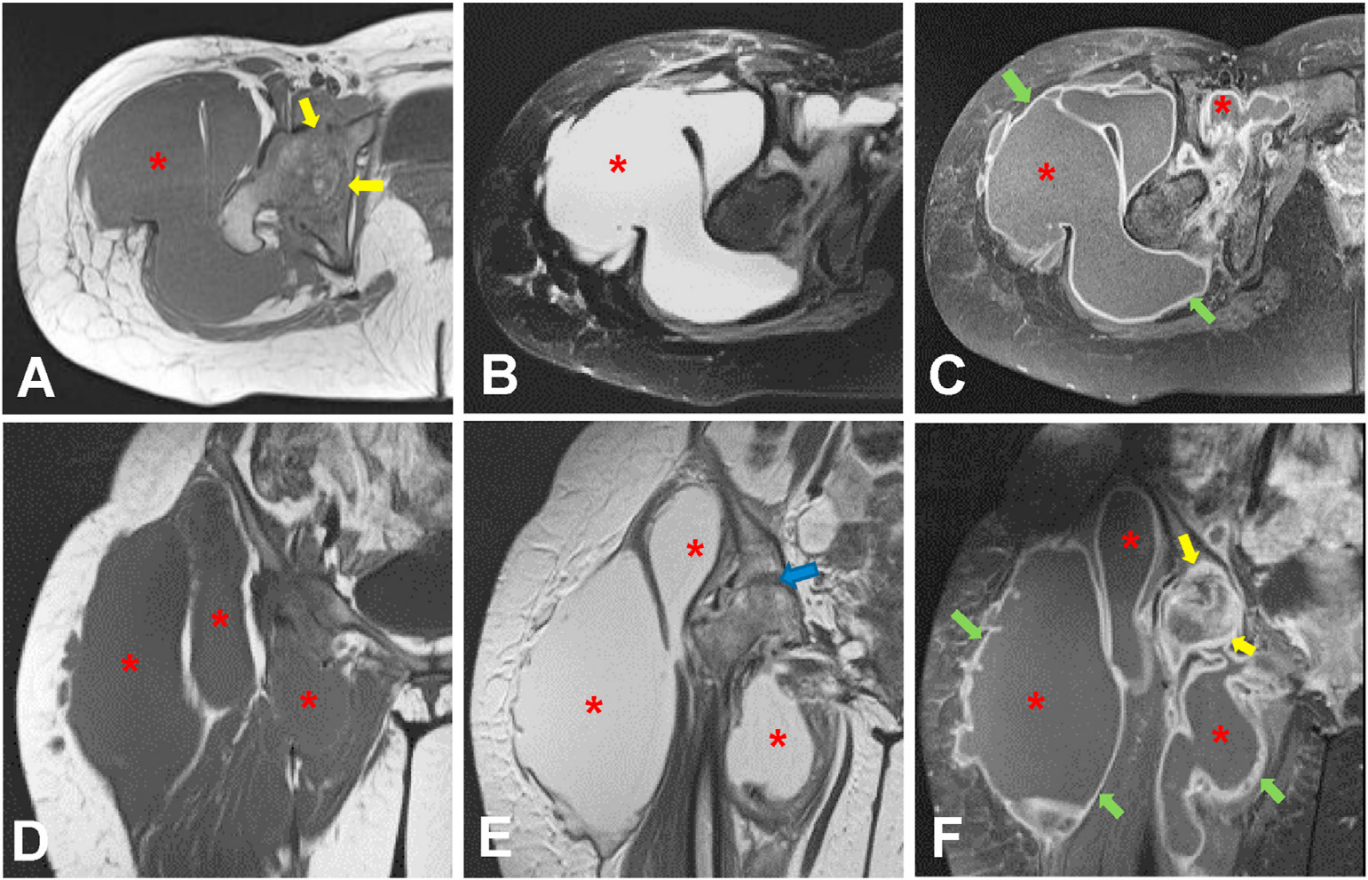
MR of the right hip: axial view in T1WI (A), T2WI fat-saturation (B) and T1WI fat-saturation with contrast (C); coronal view in T1WI (D), T2WI (E) and T1WI fat-saturation with contrast (F). There is a severe joint space loss of the hip (blue arrow) with destruction, edema and abnormal enhancement of the femoral head and acetabulum (yellow arrows). Multifocal periarticular fluid collections (asterisk) are identified, the largest one is at the lateral side of the proximal thigh. Post-contrast imaging shows thin and regular wall (green arrows) extending from the right hip joint to the midthigh.

An AP radiograph of the right femur was obtained, showed a large lobulated soft tissue mass on the lateral side of the proximal femoral region and was assessed as a soft tissue tumor (Figure 2). However, there was also central erosion and destruction of the femoral head as well as the acetabular roof. Contrast-enhanced MR showed a large rim-enhancing complex cystic mass with multiple septations and thin walls at the lateral side of the proximal femoral region, superficial to the bone, measuring 18,3 cm x 10,6 cm x 8,7 cm. The lesion was extending to the right gluteal region. However, there was also a small fluid collection with a similar pattern noted at the anteromedial part of the right hip. Enhancing bone marrow edema with an erosion of the right hip joint was also identified. Based on the enhancement pattern, there was a connection between the large cystic mass with the right hip lesion (Figure 3).

Laboratory test showed microcytic hypochromic anemia, increased white blood cells (WBC) count, (15.150/μl, normal range: 5.000–10.000/μl), increased c-reactive protein (CRP) (149.3 mg/L, normal value < 5.0 mg/L), increased erythrocyte sedimentation rate (ESR) (130 mm, normal value < 20 mm) and increased lactate dehydrogenase (LDH) (247 U/L, normal range 135–214 U/L). Other laboratory findings were unremarkable.

### 2.2 Differential diagnosis

The large lobulated cystic mass may be consistent with a large abscess formation, and its connection with the abnormality of the right hip may support the diagnosis of chronic infection. Other potential diagnoses related to fluid-filled mass in musculoskeletal lesions were true cystic lesions (such as seroma) and cystic-appearing solid neoplasm (myxofibrosarcoma and myxoid liposarcoma that contain high mucin component).

### 2.3 Treatment

The patient underwent open drainage surgery and excisional biopsy and the specimens were sent to the Pathology Department.

### 2.4 Outcome and follow up

**Figure 4:**
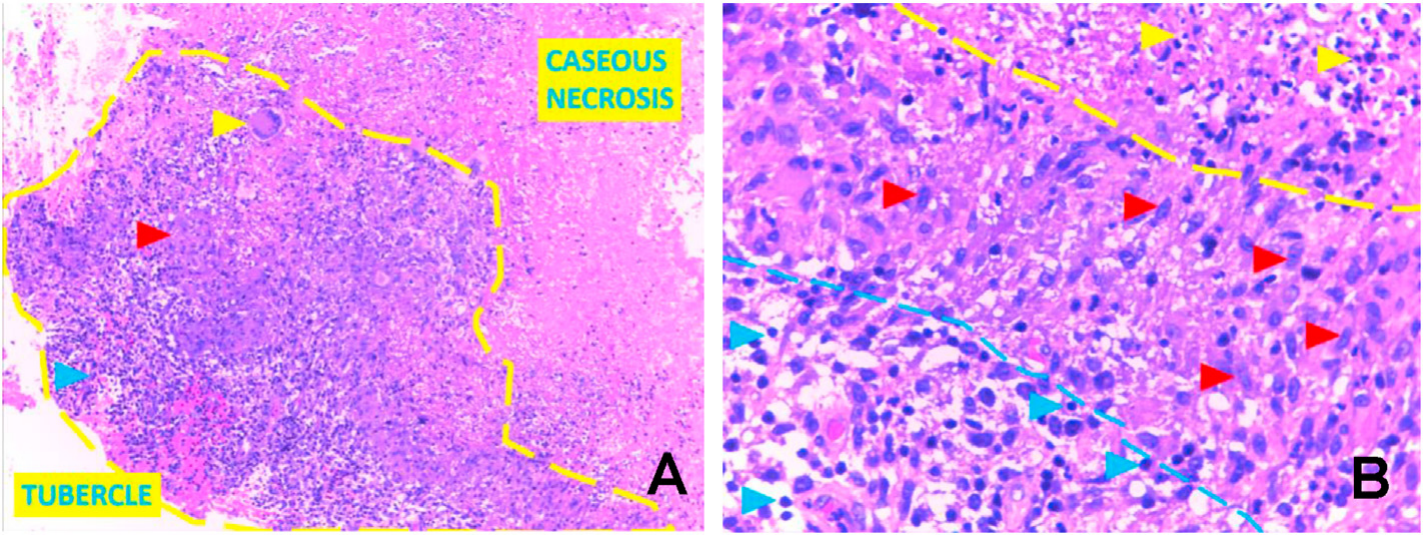
Histopathologic examination: hematoxylin-eosin staining in 100x magnification (A) and 400x magnification (B) showed the main cellular components of tubercles are epithelioid cells (red arrow), multinuclear giant cells with horseshoe arrangement (yellow arrow) and lymphocytes (blue arrow) demarcated by caseous necrosis area.

**Figure 5:**
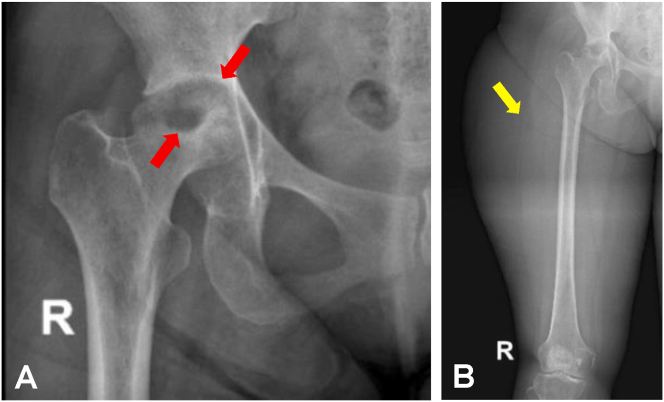
Follow-up right hip (A) dan femoral (B) radiograph after six months of anti-tuberculosis therapy. There was remaining disorganization of the hip joint with a reparative sclerotic area (red arrows) and significant reduction of soft tissue mass at the proximal thigh (yellow arrow). There is no progressive joint destruction.

The histopathology examination showed chronic granulomatous inflammation caused by tuberculous infection (Figure 4). The patient received anti-tuberculosis drugs (first-line anti-tuberculosis agents) for 9 months and the symptoms ameliorate. Six months after the anti-tuberculosis drug treatment began, the patient underwent a follow-up hip radiograph which showed remaining right hip destruction with a significant reduction of soft tissue swelling at the proximal right thigh (Figure 5). The laboratory test showed normal findings.

## 3 Discussion

Joint destruction with adjacent tumor-like mass has several differential diagnoses, including tuberculous septic arthritis, malignant soft tissue tumor, and pyogenic septic arthritis. Their clinical appearance may look similar, especially with the formation of a large painful mass. It becomes important to differentiate infection from the formation of tumor-like abscess from malignant soft tissue tumor, as they have completely different therapy. Malignant soft tissue tumors should be evaluated with core biopsy so that the clinicians can give appropriate chemotherapy, whereas infection would need immediate abscess drainage, antibiotics and debridement procedures. Delayed management may cause further destruction of the joint and results in irreversible disability, and worsened prognosis.

The potential pitfall in the radiographic interpretation, in this case, is the large size of the soft tissue mass, which may divert the reader's attention from the hip joint abnormality. Another potential misinterpretation is whether these two findings are two separate entities or whether they are associated. Without MR imaging, this can be quite challenging since the extent of the mass is quite huge compared to the degree of hip joint destruction.

Tuberculous septic arthritis is a chronic disease with long progressive clinical and radiological changes. Saraf SK et al [2] has introduced the stages of tuberculous septic arthritis: synovitis, early arthritis, and advanced arthritis with or without subluxation/dislocation. At the earliest stage, the synovitis stage shows the haziness of articular margin and rarefaction followed by periarticular bone erosions but preserved joint space. At the advanced stage, joint space becomes markedly narrowed with the shortening of the limb. It is important to define the stage of tuberculous septic arthritis to determine the appropriate management and the prognosis. We could classify the patient as advanced tuberculous septic arthritis with subluxation as it showed gross destruction and disorganization of hip joint space. Para articular bone marrow edema is also very prominent with thickened and enhanced joint capsule.

Fluid-filled mass in the musculoskeletal system may have several differential diagnoses, including true cystic lesions (abscess and seroma) and cystic-appearing solid neoplasm (myxofibrosarcoma and myxoid liposarcoma that contain high mucin component) [6]. Seroma could be characterized as an abscess-like appearance, but the seroma usually has a very thin imperceptible wall that occurs at the site of the previous procedure, whereas abscess can occur at any site of infection, with a thicker wall compared to the seroma wall [7]. On the other hand, cystic-appearing solid neoplasm like myxofibrosarcoma and myxoid liposarcoma may show cystic-appearance necrotic area, but the solid component will show marked heterogeneous enhancement [6]. Based on MR findings in the presented case, direct communication of pathological enhancement between the abscess and the hip joint was observed. The abscess was located more inferior to the hip joint. Erect position may result in a caudal extension of the abscess due to gravitational force. Similarly, the phenomenon is also noted in the tuberculous spondylitis, where the abscess formation is larger at the level inferior to the site of infection, and the size of the abscess is relatively large out of proportion to the degree of bone and joint destruction [8].

Both cystic appearing solid neoplasm and tuberculous septic arthritis may show the same painful mass-forming lesion, with no apparent local infection signs such as redness or warmth on the lump, but MR imaging and blood examination may help to differentiate them. Cystic-appearing solid neoplasm may show heterogeneous signal intensity with inhomogeneous enhancement but without increased leukocyte and CRP level, whereas tuberculous abscess formation will show thin and smooth wall enhancement with the increased number of leukocytes and CRP. Therefore, tuberculous abscess formation is more likely than cystic-appearing solid neoplasm in this patient.

Pyogenic septic arthritis can affect all age groups with no specific range of age. The most common pathogen is *Staphylococcus aureus*. As the pathogen produces proteolytic enzymes, pyogenic septic arthritis usually has an acute onset, more rapid, and more progressive [1]. It also shares some similar clinical and imaging characteristics with tuberculous septic arthritis. Hong SH et al [4] showed both tuberculous and pyogenic septic arthritis cause bone erosion, marrow signal intensity abnormality, and rim-enhancing abscess. Increased leukocyte and CRP levels are found in both pyogenic and tuberculous arthritis.

MR imaging and clinical manifestation may help differentiate pyogenic from tuberculous septic arthritis. As *M. tuberculosis* does not produce proteolytic enzymes like pyogenic pathogens, it shows less inflammation process and relative joint space preservation compared to pyogenic septic arthritis which is characterized by a more aggressive course, prominent inflammation, and progressive joint space loss [4]. The most significant imaging characteristic to differentiate tuberculosis from the pyogenic abscess is the abscess wall. The tuberculous abscess usually has a thin and smooth wall, whereas a pyogenic abscess had a thick, irregular, and nodular wall. From the clinical perspective, pyogenic septic arthritis usually shows an acute onset and more prominent local inflammation signs such as redness and warmth of the lump, whereas tuberculous septic arthritis shows less inflammation process forming “cold abscess”. As this patient showed no significant local inflammation on the right thigh with a chronic onset of the disease and a well-defined thin and smooth wall of abscess in MR finding, therefore the condition was more likely diagnosed as tuberculous septic arthritis.

The patient had open drainage surgery and excisional biopsy, and histopathology examination showed chronic granulomatous inflammation caused by tuberculous infection. Histopathology examination and good response of clinical condition after the anti-tuberculosis drug combination administration made the diagnosis of tuberculous septic arthritis in this patient.

## 4 Conclusion

The presented case was confirmed as tuberculous septic arthritis with large soft-tissue abscess formation. Tuberculous septic arthritis should be considered as one of the differential diagnoses in slow-progressing monoarticular joint pain, especially in endemic countries. As the disease progresses slowly and may cause irreversible joint destruction, early diagnosis is crucial to improve the patient outcome. MR imaging, combined with the radiograph and clinical information, played an important role in the diagnosis of tuberculous septic arthritis and differentiation to pyogenic septic arthritis or other cystic-appearing neoplasms.

## Declarations

### Author contribution statement

All authors listed have significantly contributed to the investigation, development and writing of this article.

### Funding statement

This research did not receive any specific grant from funding agencies in the public, commercial, or not-for-profit sectors.

### Data availability statement

Data will be made available on request.

### Declaration of interests statement

The authors declare no conflict of interest.

### Additional information

No additional information is available for this paper.

**Learning Points**

• The case presented shows variability of clinical symptom and imaging appearance, ranging from asymptomatic with the radiographic presentation could mimicking other abnormalities such as pyogenic septic arthritis and soft tissue tumor lesion.
• The patient with monoarticular joint pain that has slow progress symptoms, especially in endemic countries, tuberculous septic arthritis should be considered as one of differential diagnosis
• MR imaging can be beneficial to differentiate pyogenic from tuberculous septic arthritis with the most significant imaging characteristic to differentiate tuberculosis from the pyogenic abscess is the abscess wall.
